# Adaptive Mechanism for Designing a Personalized Cranial Implant and Its 3D Printing Using PEEK

**DOI:** 10.3390/polym14061266

**Published:** 2022-03-21

**Authors:** Syed Hammad Mian, Khaja Moiduddin, Sherif Mohammed Elseufy, Hisham Alkhalefah

**Affiliations:** Department of Industrial Engineering, College of Engineering, King Saud University, P.O. Box 800, Riyadh 11421, Saudi Arabia; khussain1@ksu.edu.sa (K.M.); sseufy@ksu.edu.sa (S.M.E.); halkhalefah@ksu.edu.sa (H.A.)

**Keywords:** 3D reconstruction, customized implant, PEEK, accuracy evaluation, finite element analysis, 3D printing

## Abstract

The rehabilitation of the skull’s bones is a difficult process that poses a challenge to the surgical team. Due to the range of design methods and the availability of materials, the main concerns are the implant design and material selection. Mirror-image reconstruction is one of the widely used implant reconstruction techniques, but it is not a feasible option in asymmetrical regions. The ideal design approach and material should result in an implant outcome that is compact, easy to fit, resilient, and provides the perfect aesthetic and functional outcomes irrespective of the location. The design technique for the making of the personalized implant must be easy to use and independent of the defect’s position on the skull. As a result, this article proposes a hybrid system that incorporates computer tomography acquisition, an adaptive design (or modeling) scheme, computational analysis, and accuracy assessment. The newly developed hybrid approach aims to obtain ideal cranial implants that are unique to each patient and defect. Polyetheretherketone (PEEK) is chosen to fabricate the implant because it is a viable alternative to titanium implants for personalized implants, and because it is simpler to use, lighter, and sturdy enough to shield the brain. The aesthetic result or the fitting accuracy is adequate, with a maximum deviation of 0.59 mm in the outside direction. The results of the biomechanical analysis demonstrate that the maximum Von Mises stress (8.15 MPa), Von Mises strain (0.002), and deformation (0.18 mm) are all extremely low, and the factor of safety is reasonably high, highlighting the implant’s load resistance potential and safety under high loading. Moreover, the time it takes to develop an implant model for any cranial defect using the proposed modeling scheme is very fast, at around one hour. This study illustrates that the utilized 3D reconstruction method and PEEK material would minimize time-consuming alterations while also improving the implant’s fit, stability, and strength.

## 1. Introduction

Skull or cranial deformities are becoming more common as a result of increased traffic accidents, tumors, and disasters, thus increasing the demand for skull reconstruction [1]. Cranial defect reconstruction is one of the most arduous tasks confronted by surgeons because of the unique shape of the skull. It is one of the most challenging surgical procedures owing to the complication of the skull’s form and the differences in the physiology of the skull. The very first phase in addressing cranial problems is to detach abnormally connected skull bones and replace them with accurate implants in order to restore their functionality [2]. According to Park et al. [3], customized implants for skull skeletal augmentation should be fitted properly to the cranial deficiency, reducing the gap between the implant and the bone, as well as matching the tapered borders with the neighboring bone boundaries. Personalized implants, when properly designed and manufactured, can significantly shorten the operation time, while also improving the correctness of the implant’s shape and geometry in relation to the patient’s anatomy. The fundamental idea behind these design strategies is to employ a cranium template to create an implant with an exterior shape that will adapt well to the skull while also distributing mechanical force effectively in the situation of an accident. Two primary design approaches that are considered for cranial defect reconstruction (depending on the intricacy of the cranial injury) are discussed below.

- Mirrored reconstruction design technique: This is based on the symmetrical nature of bones, and is more suitable for the treatment of unilateral skull damage [4,5,6,7]. The unhealthy or tumorous region is removed and replaced by the healthy opposite side in this technique. The precise execution of the mirroring approach is not simple, as it necessitates many manual operations, including the determination of the symmetry plane, the segregation of the healthier portion relating to the lesion, and the tweaking of the generated patch on the deficient region of the skull [8]. One of the major drawbacks of mirror-image reconstruction technique is that it cannot be used in asymmetrical body regions. If there is tumor in the center of the skull, the mirror-image design technique cannot provide the healthy opposite side.
- Anatomical reconstruction design approach: This is a curve-based surface manipulation and modification method in which two end curves of the resected regions are chosen and a guiding curve is employed for anatomical bone regeneration [9]. The fundamental benefit of the anatomical reconstruction technique is that it could be employed in both symmetrical and asymmetrical regions; nevertheless, in highly contoured parts, it requires technical competence. According to Moiduddin et al. [10], in mandible reconstruction, the anatomical reconstruction technique is more efficacious than the mirroring technique, resulting in less variation from the reference bone.

The introduction of Computer-Aided Design (CAD) and Computer-Aided Manufacturing (CAM) technologies supplanted the preceding processes that relied on manual shaping [11] and casting [12,13], allowing novel materials to be used in implant manufacturing, resulting in superior quality and enhanced postoperative results. Until recently, autogenous bone transplantation has been the most popular method of fixing skull abnormalities because it has fewer problems such as infection, aggressive foreign body reaction, soft tissue, and skin damage, etc. [14]. However, the utilization of autologous bone repair in the case of large and complicated lesions is constrained due to the scarcity of donors. As a result, there is a drive for implants made of alternative materials. Other developments in implant industries are being leveraged by recent advances in CAD systems and three-dimensional (3D) printing technologies. Patient-specific implants can now be 3D printed from a range of polymer, ceramic, or metal components [15].

Three-dimensional printing has revolutionized the manufacturing business—particularly medical, aerospace, and automobile manufacturing, construction, and so on—with its groundbreaking material deposition technique [16,17]. The possibilities of 3D printing in the healthcare industry have substantially improved the ability to build components with complex geometries using medical imaging data, which would be unachievable through traditional approaches [18]. Furthermore, 3D printing reduces the patient’s surgery time and discomfort by avoiding surgery revisions. However, there are some limits when it comes to the employment of 3D printing to fabricate bespoke implants. For instance, it is currently unclear which material produces the greatest results in cranioplasty [19]. The neurosurgeon’s choice of materials has frequently been influenced by availability, regulation, expense, and experience [15,20]. A variety of biomaterials have been employed in the fabrication of cranioplasty implants. For instance, polymethyl methacrylate (PMMA) was commonly employed because of its biocompatibility and inexpensive cost; however, it created heat during polymerization and did not chemically bind to adjacent tissue [21]. Calcium phosphate and hydroxyapatite are osteoconductive, and facilitate osteointegration, but their brittleness made them prone to breaking [22,23,24]. The most frequent biomaterials for implant reconstructions have been titanium (Ti) alloys. They offer numerous advantages, such as superior biocompatibility, great mechanical qualities, osseointegration abilities, high corrosion and wear resistance, and so on [25,26]. However, Ti and its alloys have certain disadvantages, including the possibility of metal ion release and subsequent osteolysis, metal corrosion, and inadequate compliance with contemporary imaging technologies [27]. Ti has a substantially higher modulus of elasticity (110 GPa) when compared to bone (14 GPa) (see Table 1). This significant disparity between the two frequently leads to implant malfunction due to stress shielding, bone resorption, and implant rupture [28,29]. When metallic implants are exposed to irradiation, they emit scattering rays that are detrimental to tissues. Economic reasons connected to manufacturing procedures (especially Electron Beam Melting, EBM, which is the most prevalent process for the production of Ti implants) have led researchers to hunt for new implant materials [30]. Researchers have switched to the examination of potential Ti substitutes in order to address the above-mentioned restrictions and reduce biological issues after implant insertion. 

An elevated thermoplastic polymer called polyetheretherketone (PEEK)—which is a partly crystalline, poly-aromatic, linear, thermoplastic material [41,42]—is by far the most plausible innovative substitute to Ti. It is an organic synthetic polymeric material with higher temperature stability above 300 °C, high mechanical strength, adaptable mass production, production possibility utilizing plastic techniques, natural radiolucency and MRI compatibility, nontoxicity, excellent chemical and sterilization tolerance, and a pure and quantifiable supply channel [41,43,44]. The relative density of PEEK is 1.3 to 1.5. The water absorption for PEEK after 24 h is 0.06 to 0.3%, whereas its water absorption at saturation was found to be 0.22 to 0.5% [45]. PEEK, unlike metals and alloys, offers great strength with a low Young’s modulus (3.6 GPa in its pure form, and 12 GPA in glass fiber-reinforced PEEK (GFR-PEEK)), which is nearer to that of human bone than Ti [41,44,46]. This characteristic may reduce stress by dispersing it in a most healthy manner, promoting bone growth and minimizing osteolysis surrounding the implant. The PEEK composite material has indeed been utilized extensively in the disciplines of orthopedics [47,48,49,50], dentistry [51,52,53,54], and other domains as a proven implant alternative in load-bearing body parts. However, there is a scarcity of information for PEEK as a cranial implant material, with only a few pieces of research emphasizing its application. Cranioplasty is a complicated procedure that may result in a number of serious side effects, prompting the need for revision surgery. Apparently, as mentioned in [55,56,57], the price of tailored PEEK implants is regarded to be excessively costly. Practitioners must be aware that the material is costly, but the cost of the material does not account for a significant amount of the total cost. For example, PEEK can be sterilized frequently and at a low cost. Other materials are less expensive, but sterilizing is exorbitant [58]. As a result, PEEK performs extremely fairly and provides favorable value for cranial implants. Likewise, the higher the rate of failure, the more changes and reoperations are required. Additional revisions imply greater expenses and much more mental anguish for the patient. Thien et al. [59] observed 12.5% cranioplasty failures with PEEK compared to 25% with Ti, indicating that PEEK is a more cost-effective option for cranial implants. 

The idea of customized implant design employing medical modeling software and fabrication using PEEK must be adopted in order to perfectly accommodate bone shapes and give a better aesthetic outcome. The purpose of this study is to investigate the fabrication of a cranial implant made of PEEK, as well as its mechanical characteristics and accuracy. The cranial implant is designed using a new procedure based on anatomical reconstruction. The method is discussed in detail, from computer tomography (CT) scans through to the final design and subsequent material extrusion-based 3D printing. This research also evaluates the performance of a 3D-printed PEEK implant in terms of strength using Finite Element Analysis (FEA), and in terms of accuracy through 3D Comparison analysis.

## 2. Proposed Methodology

**Figure 1:**
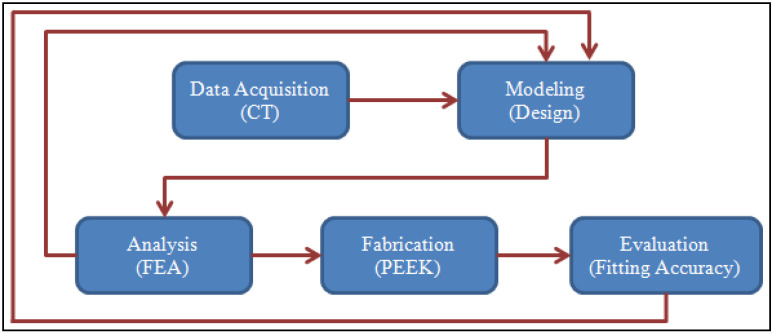
Deriving a cranial implant using a hybrid system.

Customized 3D cranial implants are essential, and they employ contemporary production methods, particularly 3D printing or additive manufacturing (AM). These approaches should incorporate the use of multiple design and analysis tools at the same time. As a result, this research develops a hybrid framework (Figure 1) that combines a CT system (as a reverse engineering or data acquisition mechanism), a design (or modeling) scheme, a computational analysis system, a fabrication process, and an accuracy assessment tool. The aim of the use of the newly established hybrid approach is to obtain an impeccable customized cranial implant irrespective of the tumor location. The designed model of the cranial implant is always well-suited to the boundary of the missing fragment in the skull, which is a unique attribute of this procedure.

### 2.1. Design and Modeling

**Figure 2:**
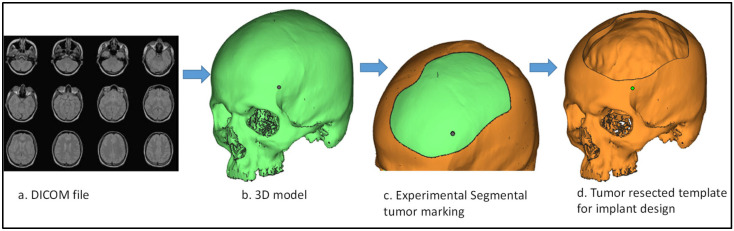
Steps involved in the reconstruction of a tumor-resected implant template. (**a**) DICOM file. (**b**) 3D model. (**c**) Experimental Segmental tumor making. (**d**) Tumor resected template for implant design.

The input component to the modeling process of the skull implant is the two-dimensional (2D) images obtained from the CT scan, as illustrated in Figure 2a. The 2D images are processed using medical modeling software, such as Mimics^®^ (Materialise, Leuven, Belgium), which converts them into a 3D structure. The hard and soft tissues are differentiated using a grayscale metric, while region growing segmentation is used to exclude unnecessary data and label the 3D model into distinct areas. Following segmentation, the region of interest is extracted and saved as a Standard Tessellation Language (STL) file for the implant’s design (Figure 2b). An experimental segmental defect (Figure 2c) is marked on a healthy skull, which acts as a template for implant design, as shown in Figure 2d. The skull template with a resected tumor is crafted to evade the inconvenience of obtaining patient and ethical committee approvals. The ultimate purpose is to communicate how to model the implant design precisely, and to replicate the characteristics of a complex injury, tumor or wound on the skull regardless of the location. Furthermore, using this approach, the healthy STL skull model can be used as a reference in order to compare the implanted skull’s fitting accuracy, which is explained in the following sections. 

**Figure 3:**
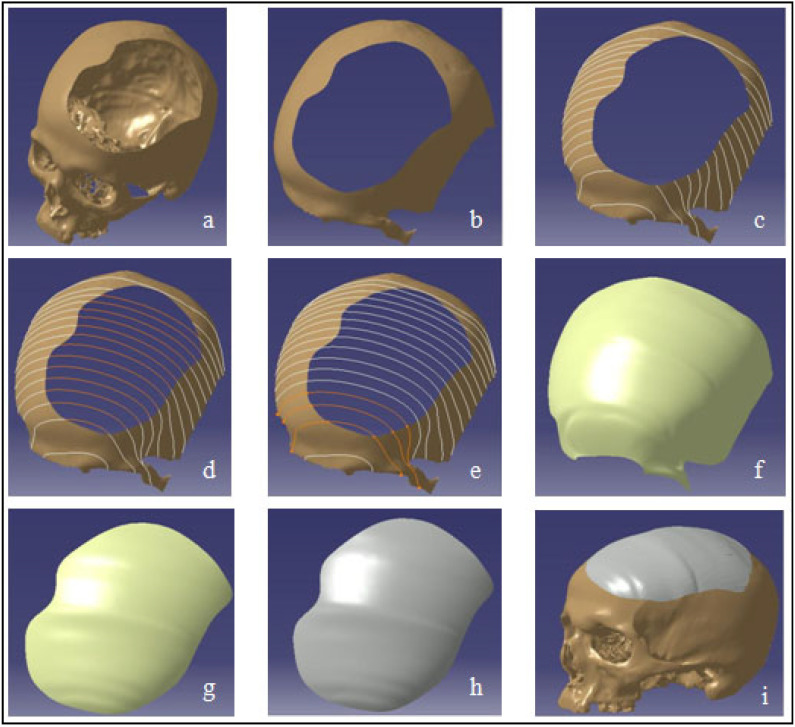
Modeling of the customized implant (**a**) asymmetrical skull defect; (**b**) identification of distorted region; (**c**) recognition of curves tangent to surface; (**d**) interpolation of curves; (**e**) surface generation; (**f**) surface acquired through curves; (**g**) splitting appropriate surface patch; (**h**) conversion of surface to part model; (**i**) implant placed on the skull.

The next task is to design a custom cranial implant model based on the asymmetrical skull regional defect using the skull template (Figure 3a). The application of interpolation spline curves is the foundation for the construction of an implant model that is adapted to a skull bone with an incomplete segment [60]. The entire modeling process is accomplished by combining different modules such as the Digitized Shape Editor, Generative Shape Design, and Quick Surface Reconstruction in Catia V5. The approach proposed is universal, and can be used regardless of the location where the skull bone loss occurs. 

The distorted region (Figure 3b) is identified for further processing using the Activate tool in the Digitized Shape Editor during the first stage of the modeling process. This stage can be skipped; however, working on a relatively smaller volume is less time-consuming and easier. As shown in Figure 3c, spline curves tangent to the surface of the skull model are then defined. The Planar Sections option is used for this reason. It computes curves by cutting a cloud of points or a mesh using planes. Because the curves are disconnected due to the resected skull hole fragment, the shape of the curves is interpolated between the existing neighboring nodes, culminating in a spline curve that passes seamlessly through all of the appropriate points (Figure 3d). For this, the Connect Curve option (in Generative Shape Design) is used, which produces a connecting curve between two curves. As a consequence, the obtained spline curves (Figure 3e) are utilized to facilitate surface patching. The surface generation process is carried out in the CATIA V5 system’s Quick Surface Reconstruction environment. The multi-section surface mechanism is chosen to build the surface patch along the curvature of the spline curves. The established surface patch is much larger than the resected skull tumor region, as depicted in Figure 3f. The redundant (or excess) surface is split into the shape of the opening on the skull by Boolean operation in order to achieve the desired form of the implant surface (Figure 3g). The transformation of the implant surface model into the part model, as shown in Figure 3h, is the final stage of the process. As can be seen in Figure 3i, the implant is precisely positioned on the defect region of the skull.

### 2.2. Computational Model and Analysis

The 3D model is corrected for small geometrical errors prior to the FEA study, such as the deletion of intersecting and overlapping triangles on both the implant and skull portion. The FEA model was chosen to examine the biomechanical stability of the custom cranial implant that has been developed [1]. The purpose is to objectively assess and simulate the behavior of the designed PEEK implant in actual working scenarios. The FEA can forecast the possibility of cracks, vulnerable spots, and malfunctions of the point of implant–bone contact in a digital setting. The ANSYS (Version 19.1, Canonsburg, Pennsylvania, United States) and Hypermesh program (Version 14.0, Altaire Hyper works, Troy, Michigan, United States) are used to perform the pre-processing, post-processing, and execution of the built FEA model. The computational model constructed consists of three elements: the Skull, the PEEK implant and the fixation screws. Table 2 lists the properties of the materials assigned to the FEA model. Different regions of the skull are given different material properties [61]. The cranium is given cortical bone characteristics, while the personalized cranial implant is assigned PEEK properties [62]. Six screws of titanium are fastened at selected reference points. 

**Figure 4:**
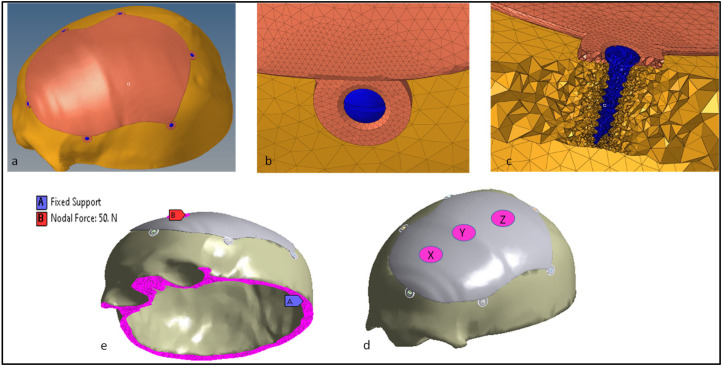
(**a**) Cranial PEEK implant on the skull model; (**b**) mesh generation; (**c**) cross-sectional view of the screw, implant and bone; (**d**) exertion of forces at the three implant regions; (**e**) representation of the fixed supports and the nodal force.

As illustrated in Figure 4a, the implant is fixed onto the skull model using six screws, whereby the contact between the plate–bone and the bone-screws is defined as bonded. The Hypermesh is applied (Figure 4b) in the establishment of the finite element (FE) model of the skull and the implant using the solid element of the tetra4 type, where the dimensions of the mesh range in size between 3 and 0.25 mm. A finer mesh is created to minimize mesh distortion and to optimize element quality, making a total of 828,683 elements and 213,805 nodes for the implant and bone portion. The mesh is then imported into ANSYS to provide the boundary and loading conditions. Figure 4c illustrates the cross-sectional view of the interface between the implant, the screw and the skull bone. A set of three circular patches of X, Y and Z are considered on the implant for loading (Figure 4d). The base of the skull is held constant for all of the configuration by fixing it at the bottom, and a static force of 50 N is exerted (Figure 4e) at each of the three regions of the implant over a small area of 225 mm^2^ [63,64]. In cranial reconstruction, the implant–bone assembly fixture and its mechanical robustness is of extreme importance for the long-tern clinical success. Several others research studies have demonstrated the FE model of skull implants with respect to different shapes, geometry, fixation devices and materials [64,65]. In this study, a computational model of a PEEK implant with a static load of 50 N is evaluated for the biomechanical responses of stress, strain, deformation, and for the factor of safety. The factor of safety is also a good measure for the acceptance of a design on the basis of its withstanding capacity for the intended load.

### 2.3. Fabrication of the Skull Model and Cranial Implant

**Figure 5:**
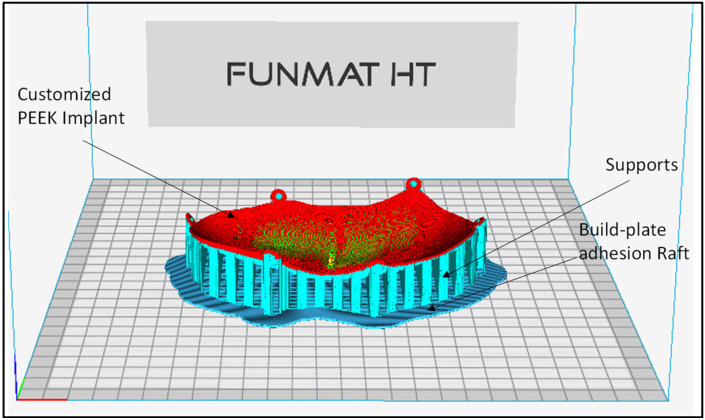
INTAMSUITE software for the orientation and support generation of the PEEK 3D model.

The fabrication of the cranial implant is initiated by the correction of errors in the STL file. Magics^®^ is employed to correct defects such as overlapping, intersecting triangles, poor corners, and other flaws. The processing of the STL file and the specification of the optimal location and orientation for fabrication are the goals of this step. Once the STL model is perfect, appropriate supports are created on the overhanging structures. In order to construct the prototype precisely and without any abnormality, the proper supports are needed. The support structures are also needed for efficient heat transfer, in order to prevent any distortion and to make it easier to fabricate overhanging sections. The slicing, orientation and support generation on the PEEK 3D model are performed using INTAMSUITE slicing software (Version 3.6.2, INTAMSYS Technology Co. Ltd, Shanghai, China) as illustrated in Figure 5. There are several build-plate adhesion types in the slicing software. In this model, a raft build-plate is utilized because it provides the added thick grid between the model and the build plate, and avoids warping effects, thus ensuring that the model better sticks to the build plate.

**Figure 6:**
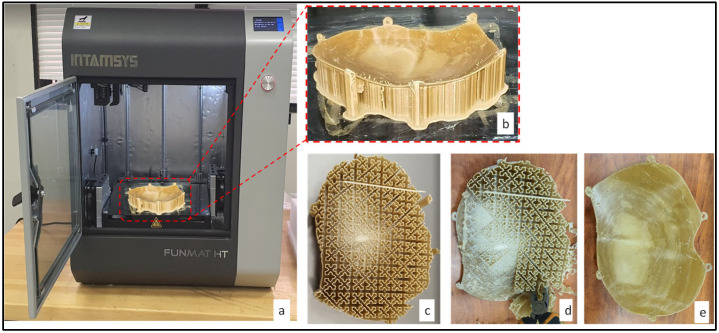
(**a**) Intamsys 3D Printer used in the fabrication of the customized (**b**) PEEK cranial Implant; (**c**) Support structures bottom view; (**d**) removal of the supports using plyers; (**e**) the PEEK cranial implant after the supports’ removal.

An INTAMSYS (Intelligent Additive Manufacturing Systems) FUNMAT HT 3D printer (INTAMSYS Technology Co. Ltd, Shanghai, China) is used for the manufacturing of the personalized PEEK cranial implant. The printer uses a fused filament fabrication (FFF) technique, which has the advantage of consuming less energy, producing more, and delivering higher tensile strength when the printing parameters are set appropriately [66,67]. FFF is amongst the most widespread and straightforward AM procedures [68]. The fabrication of 3D-printed objects is controlled by many parameters in this technology. As a result, the selection of the appropriate parameters for the fabrication of any component is essential. The process parameters used for the fabrication of the PEEK cranial implant are provided in Table 3. The printer is equipped with both the heated build plate (reaching up to 160 °C) and a build chamber (reaching up to 90 °C). The high-temperature extruder (450 °C) is used in the extrusion of high-temperature PEEK material. The infill parameter in the slicing software is set to 100% in order to provide a completely solid structure. The post-processing of the PEEK cranial implant includes the removal and pulling away of the support structures. The wall of the support structures is made less dense for easy removal. Cutting and gripping pliers are used with protective gloves to access the underneath of the supports, and to carefully bend them upwards to remove them. The time taken to remove the supports is approximately 30 to 40 min. Figure 6a, b illustrates the 3D-printed customized PEEK implant with supports (Figure 6c), where the supports are easily removed manually using plyers (Figure 6d) to obtain the final PEEK implant (Figure 6e).

**Figure 7:**
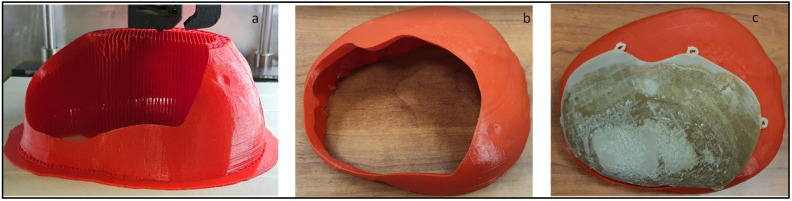
(**a**) 3D printed skull model of the ABS material with supports, (**b**) ABS skull model after the removal of the supports, and (**c**) the rehearsal and fitting evaluation of the PEEK cranial implant on the ABS skull model.

Similarly, skull model is fabricated (Figure 7a) by employing the same INTAMSYS 3D Printer using acrylonitrile butadiene styrene (ABS) material for the purposes of evaluation and testing. The ABS skull model after the removal of supports (Figure 7b) and the final PEEK implant are tested for custom fitting and rehearsal evaluation, as shown in Figure 7c. 

### 2.4. Accuracy Assessment

**Figure 8:**
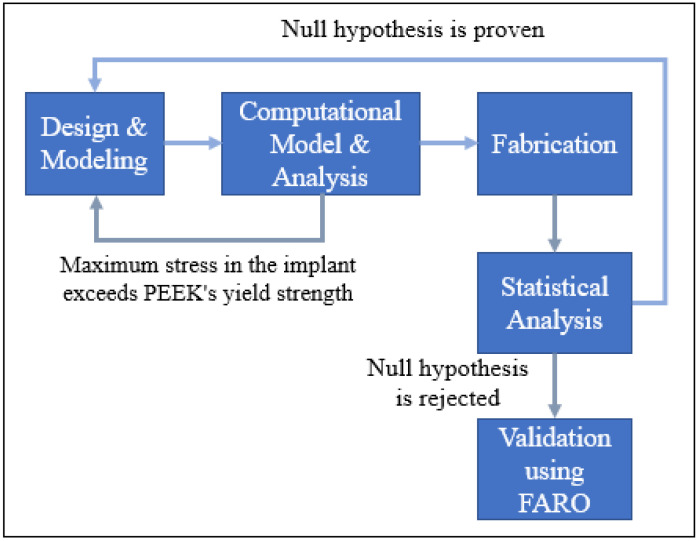
Scenarios for redesigning the implant.

The Intamsys PEEK implant with a polymer skull structure is used for the planning and fitting evaluation. According to Wyleżoł et al. [60], an overall inspection of the implant shape should be performed by professionals prior to the fabrication and implanting. As a result, the implant’s placement on the skull and cosmetic effects are evaluated using a visual analogue score (VAS) of 1 to 5 (1, bad; 2, average; 3, satisfactory; 4, fine; 5, excellent) [69]. Five health professionals and five research experts from the surgical unit are provided with the implanted skull. A PEEK implant model is produced for the specified design and delivered to the advisors for approval. Every analyst is encouraged to grade the implant independently by looking at cranial uniformity, connectivity, and visual attractiveness. The model assembly is then evaluated using a visual score ranging from 1 to 5 by all of the reviewers. The average aesthetic score is then calculated. In this study, the null hypothesis and alternative hypothesis are investigated for statistical analysis [70,71]. The null hypothesis asserts that the median aesthetic score is less than or equal to 3 (H_0_: *µ* ≤ 3), whereas the alternative hypothesis asserts that it is larger than 3 (H_a_: *µ* > 3) [18]. Finally, the implant is validated using the FARO if the null hypothesis is rejected. Otherwise, the implant is redesigned and produced again if the null hypothesis is proven, as illustrated in Figure 8. 

**Figure 9:**
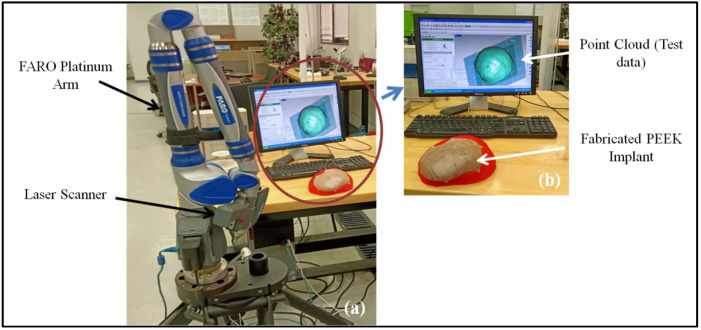
(**a**) Scanning system; (**b**) acquired point cloud data.

The fitting precision of the PEEK implant is also quantified in order to ascertain deviation from the reference (or actual) skull shape. It is estimated utilizing a 3D comparison analysis in Geomagics Control^®^ (Version 2014, 3D Systems Inc., Cary, NC, USA) [72]. It is considered to be amongst the most comprehensive and structured approaches for the interactive assessment of surface variations between the real object and the reference CAD model [73]. The test data is acquired after the custom-made cranial implant is built and fixed on the skull. The scanning is gathered with the laser scanner on the Faro Platinum arm (FARO, Lake Mary, FL, USA), as illustrated in Figure 9a. The captured point cloud data (shown in Figure 9b) is exploited as test data in the 3D comparison assessment.

The initial stage in the 3D comparison analysis is to classify the test object and the reference CAD model. The outside surface of the reconstructed skull (or the skull with the implant) is digitized and imported as an STL model into Geomagics Control^®^. The outer surface (test data) of the skull is inspected because the personalized cranial implant is built depending on the outside contour of the skull. The CAD model of a healthy (or the original) skull is employed as a reference. The quality of the reconstructed skull is then examined using a 3D comparison analysis. The test data (collected point cloud) is aligned with the reference CAD model using the best fit alignment technique. The best-fit alignment approach is used to ensure that the test and reference entities are in the same coordinate system. The average deviation in the positive direction is used to assess the implant’s accuracy. The average deviation statistic is chosen because it indicates the average deviation in outward direction, thus approximating the gap between the remodeled skull (or the tailored implant) and the original skull.

**Figure 10:**
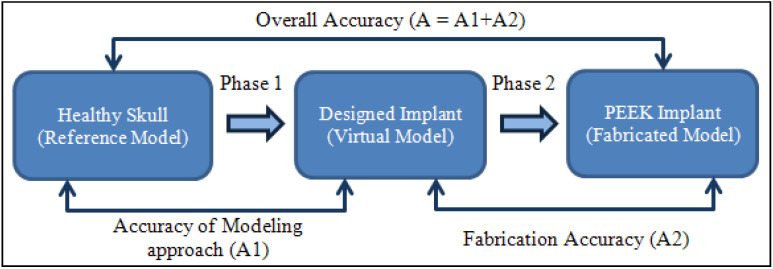
Procedure to estimate the fitting accuracy of the implant.

**Figure 11:**
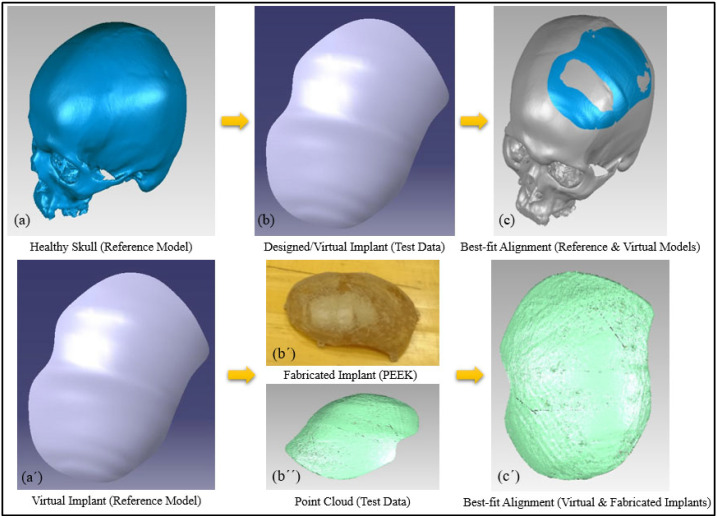
Computation of implant’s overall fitting accuracy. (**a**–**c**) Phase 1: Accuracy of the modeling approach; (**a′**,**b′**,**b″**,**c′**) Phase 2: Fabrication accuracy.

As shown in Figure 10, the accuracy evaluation is divided into two phases. The virtual model of the designed implant is compared to the original skull in phase one. This determines the amount of error (or accuracy) caused by the modeling method used. The fabricated PEEK implant is examined in phase 2 in relation to the virtual model of the designed implant. This assists in the quantification of the fabrication error. The cumulative error can be derived by adding the errors from Phases 1 and 2, as depicted in Figure 11. This also provides the implant’s overall fitting accuracy.

## 3. Results and Discussions

**Figure 12:**
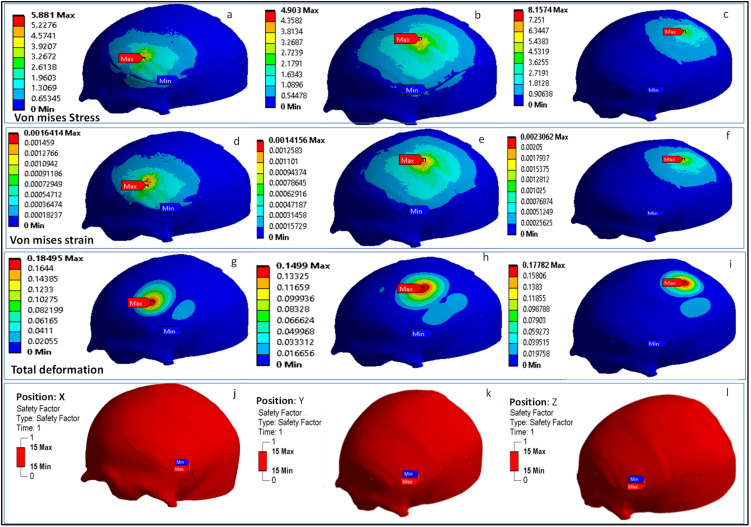
FEA results illustrating: (**a**–**c**) Von Mises stress at the point, (**d**–**f**) Von mises strain, (**g**–**i**) total deformation, and (**j**–**l**) the factor of safety at points X, Y and Z.

The results of the computational model are illustrated in Figure 12, displaying the Von Mises stress, Von Mises strain, total deformation, and safety factor at three points: X, Y and Z. The ANSYS FE solver with a static framework was chosen. The time taken to complete the computational model was approximately 450 s using the HP Z800 workstation. The results of the FEA are also used to provide input for the modeling process. For example, if the maximum stress in the implant exceeds the PEEK’s yield strength, the implant’s thickness should be modified.

The analysis results showed the largest Von Mises stress of 8.15 MPa at the patch Z, and the lowest—4.90 MPa—at patch Y, which is well below the yield point (the failure criterion) of the material, which is 99.9 MPa. The maximum Von Mises strain was found to be 0.002 (2000 με) at patch Z, and lowest was found to be 0.0014 (1400 με) at patch Y, which is also well below the assumed mechanical strain limit of humans, i.e., 3000 με [74]. In addition, studies stated that strains of more than 3500 με lead to bone resorption, and strains over the limit of 4000 με lead to bone fracture [75,76]. The implant deformation—with a maximum of 0.18 mm at patch X and a minimum of 0.14 mm at patch Z—is also within the millimeter domain. The FEA results also indicate that the factor of safety for the PEEK implant is reasonably high, suggesting safe performance under high loading. The analysis results of our computational model represent favorable nominal values which are sufficient for optimality analysis and within the scope in comparison to other cranial studies [64]. In addition, the values obtained from the FEA results in this work match those of other research studies using similar kinds of PEEK cranial implant [77]. The computational model setup used in this study illustrates an effective and relatively simple way to evaluate cranial reconstruction while avoiding complex design setup and computational expenses.

In the implant fitting evaluation, the mean aesthetic score of 3.47 out of 5 (*n* = 10) was achieved, which signifies favorability in aesthetic achievement. With a *p*-value of less than 0.05, the result was statistically significant. The hypothesis testing was carried out using a one-sample *t*-test in Minitab Statistical Software (Minitab 21, MINITAB Ltd., United Kingdom). The null hypothesis was disproved because the *p*-value (=0.003) was less than the significance level (*α*) of 0.05, and it can be claimed that the average aesthetic score was larger than 3, showing expert contentment and assurance. This emphasizes the fact that the implant design allows for a good fit on the skull and a pleasing cosmetic appearance.

**Figure 13:**
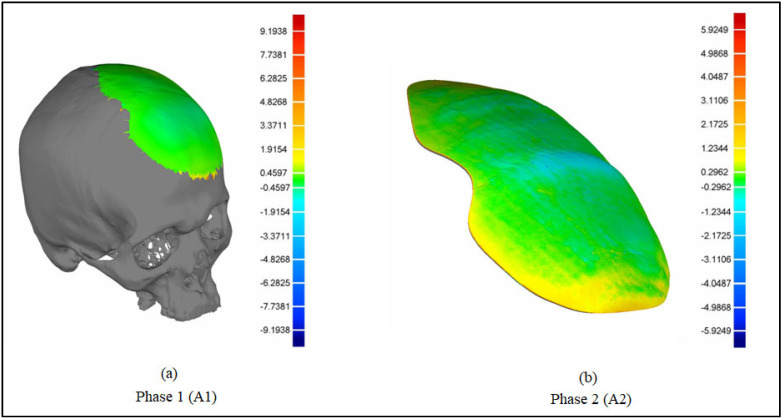
Deviation in the outward direction. (**a**) Healthy skull and the designed implant (virtual model); (**b**) designed implant (virtual model) and the fabricated implant.

**Figure 14:**
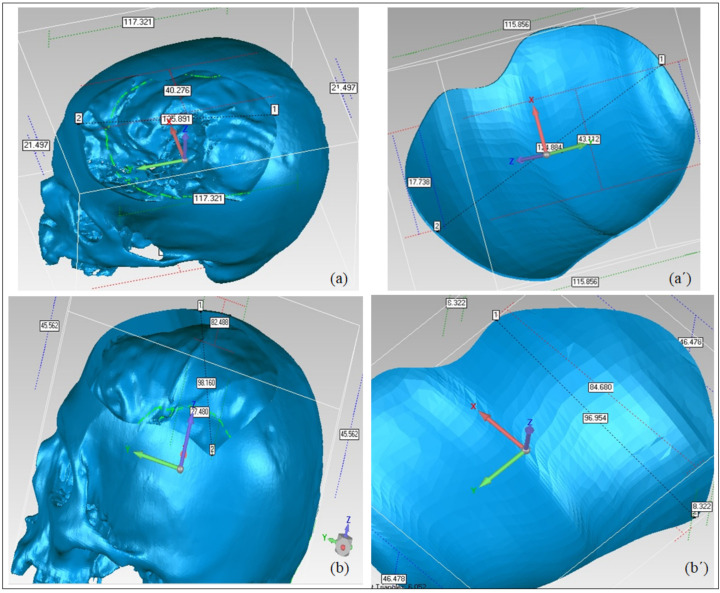
Gap Analysis between the implant and the skull. (**a**) Defect length in the Y-direction; (**a’**) implant length in the Y-direction; (**b**) defect width in the X-direction; (**b’**) implant width in the X-direction.

Figure 13a,b demonstrates the results of the 3D Comparison analysis for the healthy skull and virtual implant model, as well as the virtual implant model and the manufactured model. The modeling approach (A1) had an accuracy of 0.0687 mm, indicating that the anatomical design approach used is quite precise, and that its perfection may be improved with further expertise and experience. Furthermore, the fabrication accuracy (A2) was estimated to be 0.5232 mm, as evidenced by the outer-direction deviation. This indicates that the implant’s overall accuracy (including both A1 and A2) is 0.5919 mm. It was rather more accurately fitted when compared to the Ti4Al6V implant, which had an overall deviation of 0.9529 mm, as reported in [18]. As a result, the repaired skull produced with the proposed design technique and PEEK material is acceptable as well as appealing. In addition to the deviation analysis, the gap analysis results are also provided in Figure 14. According to the gap analysis, the distance between the implant and the skull is reasonably low in both the X and Y directions. For instance, the average implant lengths in the X and Y directions are 97.11 and 124.97 mm, respectively. The average length of the cavity (formed after removing the flaw) is 98.02 and 125.69 mm, in the X- and Y-directions respectively. This means that there is a gap of 0.91 mm and 0.71 mm in the X- and Y-direction, respectively, which is less than 1 mm, thus implying a greater fit.

## 4. Conclusions

The surgical team faces a challenging task when it comes to the reconstruction of the skull’s bones. The major aspects in cranial reconstruction are the implant design and the selection of a suitable material, owing to the variety of design methods and materials available. The optimum design strategy and material should result in a compact, easy-to-fit, robust implant that achieves the best cosmetic and functional results irrespective of the defective location. The customized implant design technique must be simple to implement and irrespective of the location of the lesion on the cranium.

The cranial implant—built using a revolutionary design technique based on anatomical reconstruction—delivered good precision, with a maximum deviation of 0.0687 mm. Similarly, the implant made of PEEK performed satisfactorily in terms of both gaps and deviations. The overall deviation of the PEEK implant was 0.5919 mm, with an average gap of less than 1 mm in both the X and Y axes, which is absolutely acceptable. Furthermore, the biomechanical analysis showed that the maximal Von Mises stress (8.15 MPa), Von Mises strain (0.002), and deformation (0.18 mm) were all remarkably low, emphasizing the implant’s load-bearing capacity.

The adopted design technique can be used as a substitute for other existing design approaches because it is also quick and precise. PEEK material can also be used as an alternative to Ti implants because it has greater fitting accuracy and enhanced load bearing capabilities. The established design technique can also be applied to enhance the effectiveness of highly complex implant designs in both symmetrical and asymmetrical body regions. The PEEK material and the personalized implant design technique used in this study can also be applied in other areas of bone surgery where implants are used for rehabilitation. Additionally, more research is essential in order to confirm the biomechanical and biological viability of the FFF-based PEEK implants.

## Figures and Tables

**Table 1:** Properties of various materials [31,32,33,34,35,36,37,38,39,40].

Material	Elastic Modulus (GPA)	Density (g/cm^3^)	Thermal Conductivity (W/m.K)	Biocompatible
Titanium alloy (Ti6Al4V)	110	4.5	7.1	Yes
Cobalt-Chromium	180–210	10	9.4	Yes
Zirconia	210	5.68	1.7–2.7	Yes
Porcelain	68.9	2.3–2.4	1.5	Yes
PMMA	3–5	1.18	0.167–0.25	Yes
PEEK	3–4	1.3–1.32	0.25–0.93	Yes
CFR-PEEK	18	1.42	0.95	Yes
GFR-PEEK	12	1.55	0.35	Yes
Cortical Bone	14	1.6–2	0.68	
Cancellous bone	1.34	0.05–0.3	0.42	
Enamel	40–83	2.6–3	0.45–0.93	
Dentin	15–30	1.79–2.12	0.11–0.96	

**Table 2:** Material properties used in the computational model [61,62].

Materials	Youngs Modulus (MPa)	Poisson’s Ratio	Yield Strength (MPa)
Cortical bone	13,700	0.3	122
PEEK implant	3738	0.4	99.9
Titanium screws	120,000	0.3	930

**Table 3:** Process parameters in the fabrication of the PEEK implant using the FUNMAT HT printer.

Description	Parameters
Printing Technology	FFF
Extruder	Single
Extruder diameter (mm)	0.4
Layer thickness ((mm)	0.15
Print speed (mm/s)	50
Filament diameter (mm)	1.75
Build adhesion type	Raft
Nozzle temperature (℃)	420
Build temperature (℃)	130
Chamber temperature (℃)	90
Nozzle used	High-temperature nozzle set

## Data Availability

The data presented in this study are available in the article.

## References

[1] Ameen W., Al-Ahmari A., Mohammed M.K., Abdulhameed O., Umer U., Moiduddin K. (2018). Design, Finite Element Analysis (FEA), and Fabrication of Custom Titanium Alloy Cranial Implant Using Electron Beam Melting Additive Manufacturing. Adv. Prod. Eng. Manag..

[2] Mandolini M., Brunzini A., Serrani E.B., Pagnoni M., Mazzoli A., Germani M. (2019). Design of a Custom-Made Cranial Implant in Patients Suffering from Apert Syndrome. Proc. Des. Soc. Int. Conf. Eng. Des..

[3] Park D.K., Song I., Lee J.H., You Y.J. (2013). Forehead Augmentation with a Methyl Methacrylate Onlay Implant Using an Injection-Molding Technique. Arch. Plast Surg.

[4] Chen X., Xu L., Li X., Egger J. (2017). Computer-Aided Implant Design for the Restoration of Cranial Defects. Sci. Rep..

[5] Kung W.-M., Tzeng I.-S., Lin M.-S. (2020). Three-Dimensional CAD in Skull Reconstruction: A Narrative Review with Focus on Cranioplasty and Its Potential Relevance to Brain Sciences. Appl. Sci..

[6] Brunzini A., Mandolini M., Manieri S., Germani M., Mazzoli A., Pagnoni M., Iannetti G., Modugno A. Orbital Wall Reconstruction by Selective Laser Sintered Mould. Proceedings of the 2017 13th IASTED International Conference on Biomedical Engineering (BioMed).

[7] Igor D., Hren N., Strojnik T., Brajlih T., Valentan B., Pogačar V., Hartner T. (2008). Applications of Rapid Prototyping in Cranio- Maxilofacial Surgery Procedures. Int. J. Biol. Biomed. Eng..

[8] Buonamici F., Furferi R., Genitori L., Governi L., Marzola A., Mussa F., Volpe Y. (2019). Reverse Engineering Techniques for Virtual Reconstruction of Defective Skulls: An Overview of Existing Approaches. Comput. -Aided Des. Appl..

[9] Harrysson O.L., Hosni Y.A., Nayfeh J.F. (2007). Custom-Designed Orthopedic Implants Evaluated Using Finite Element Analysis of Patient-Specific Computed Tomography Data: Femoral-Component Case Study. BMC Musculoskelet. Disord..

[10] Moiduddin K., Al-Ahmari A., Nasr E.S.A., Mian S.H., Al Kindi M. (2016). A Comparison Study on the Design of Mirror and Anatomy Reconstruction Technique in Maxillofacial Region. Technol. Health Care.

[11] Chee Kai C., Siaw Meng C., Sin Ching L., Seng Teik L., Chit Aung S. (2000). Facial Prosthetic Model Fabrication Using Rapid Prototyping Tools. Integr. Mfg Syst..

[12] Bhargava D., Bartlett P., Russell J., Liddington M., Tyagi A., Chumas P. (2010). Construction of Titanium Cranioplasty Plate Using Craniectomy Bone Flap as Template. Acta Neurochir.

[13] Dumbrigue H.B., Arcuri M.R., LaVelle W.E., Ceynar K.J. (1998). Fabrication Procedure for Cranial Prostheses. J. Prosthet. Dent..

[14] Abdel-Haleem A.K., Nouby R., Taghian M. (2011). The Use of the Rib Grafts in Head and Neck Reconstruction. Egypt. J. Ear Nose Throat Allied Sci..

[15] Bonda D.J., Manjila S., Selman W.R., Dean D. (2015). The Recent Revolution in the Design and Manufacture of Cranial Implants: Modern Advancements and Future Directions. Neurosurgery.

[16] Khorasani A., Gibson I., Veetil J.K., Ghasemi A.H. (2020). A Review of Technological Improvements in Laser-Based Powder Bed Fusion of Metal Printers. Int. J. Adv. Manuf Technol..

[17] Ali M.d.H., Issayev G., Shehab E., Sarfraz S. (2022). A Critical Review of 3D Printing and Digital Manufacturing in Construction Engineering. Rapid Prototyp. J..

[18] Mian S.H., Moiduddin K., Abdo B.M.A., Sayeed A., Alkhalefah H. (2022). Modelling and Evaluation of Meshed Implant for Cranial Reconstruction. Int. J. Adv. Manuf Technol..

[19] Rukskul P., Suvannapruk W., Suwanprateeb J. (2019). Cranial Reconstruction Using Prefabricated Direct 3DP Porous Polyethylene. Rapid Prototyp. J..

[20] Yadla S., Campbell P.G., Chitale R., Maltenfort M.G., Jabbour P., Sharan A.D. (2011). Effect of Early Surgery, Material, and Method of Flap Preservation on Cranioplasty Infections: A Systematic Review. Neurosurgery.

[21] Marchac D., Greensmith A. (2008). Long-Term Experience with Methylmethacrylate Cranioplasty in Craniofacial Surgery. J. Plast. Reconstr. Aesthetic Surg..

[22] Rupprecht S., Merten H.-A., Kessler P., Wiltfang J. (2003). Hydroxyapatite Cement (BoneSourceTM) for Repair of Critical Sized Calvarian Defects—an Experimental Study. J. Cranio-Maxillofac. Surg..

[23] Pang D., Tse H.H., Zwienenberg-Lee M., Smith M., Zovickian J. (2005). The Combined Use of Hydroxyapatite and Bioresorbable Plates to Repair Cranial Defects in Children. J. Neurosurg..

[24] Kanno Y., Nakatsuka T., Saijo H., Fujihara Y., Atsuhiko H., Chung U., Takato T., Hoshi K. (2016). Computed Tomographic Evaluation of Novel Custom-Made Artificial Bones, “CT-Bone”, Applied for Maxillofacial Reconstruction. Regen. Ther..

[25] De Viteri V.S., Fuentes E. (2013). Titanium and Titanium Alloys as Biomaterials.

[26] Trevisan F., Calignano F., Aversa A., Marchese G., Lombardi M., Biamino S., Ugues D., Manfredi D. (2018). Additive Manufacturing of Titanium Alloys in the Biomedical Field: Processes, Properties and Applications. J. Appl. Biomater. Funct. Mater..

[27] Tengvall P., Lundström I. (1992). Physico-Chemical Considerations of Titanium as a Biomaterial. Clin. Mater..

[28] Huiskes R., Ruimerman R., van Lenthe G.H., Janssen J.D. (2000). Effects of Mechanical Forces on Maintenance and Adaptation of Form in Trabecular Bone. Nature.

[29] Lee W.-T., Koak J.-Y., Lim Y.-J., Kim S.-K., Kwon H.-B., Kim M.-J. (2012). Stress Shielding and Fatigue Limits of Poly-Ether-Ether-Ketone Dental Implants. J. Biomed. Mater. Res. B Appl. Biomater..

[30] Agapovichev A., Sotov A., Kokareva V., Smelov V. (2018). Possibilities and Limitations of Titanium Alloy Additive Manufacturing. MATEC Web Conf..

[31] Rahmitasari F., Ishida Y., Kurahashi K., Matsuda T., Watanabe M., Ichikawa T. (2017). PEEK with Reinforced Materials and Modifications for Dental Implant Applications. Dent. J..

[32] Dondi M., Ercolani G., Marsigli M., Melandri C., Mingazzini C. (1999). The Chemical Composition of Porcelain Stoneware Tiles and Its Influence on Microstructure and Mechanical Properties. InterCeram: Int. Ceram. Rev..

[33] Brockett C.L., Carbone S., Fisher J., Jennings L.M. (2017). PEEK and CFR-PEEK as Alternative Bearing Materials to UHMWPE in a Fixed Bearing Total Knee Replacement: An Experimental Wear Study. Wear.

[34] Grimal Q., Laugier P. (2019). Quantitative Ultrasound Assessment of Cortical Bone Properties Beyond Bone Mineral Density. IRBM.

[35] Zioupos P., Cook R.B., Hutchinson J.R. (2008). Some Basic Relationships between Density Values in Cancellous and Cortical Bone. J. Biomech..

[36] Gradl R., Zanette I., Ruiz-Yaniz M., Dierolf M., Rack A., Zaslansky P., Pfeiffer F. (2016). Mass Density Measurement of Mineralized Tissue with Grating-Based X-Ray Phase Tomography. PLoS ONE.

[37] Lancaster P., Brettle D., Carmichael F., Clerehugh V. (2017). In-Vitro Thermal Maps to Characterize Human Dental Enamel and Dentin. Front. Physiol..

[38] Feldmann A., Wili P., Maquer G., Zysset P. (2018). The Thermal Conductivity of Cortical and Cancellous Bone. Eur Cell Mater..

[39] Rivière L., Caussé N., Lonjon A., Dantras É., Lacabanne C. (2016). Specific Heat Capacity and Thermal Conductivity of PEEK/Ag Nanoparticles Composites Determined by Modulated-Temperature Differential Scanning Calorimetry. Polym. Degrad. Stab..

[40] Properties: Zirconia—ZrO2, Zirconium Dioxide. https://www.azom.com/properties.aspx?ArticleID=133.

[41] Gowda E.M., Iyer S.R., Verma K., Murali Mohan S. (2018). Evaluation of PEEK Composite Dental Implants: A Comparison of Two Different Loading Protocols. J. Dent. Res. Rep..

[42] Sharma N., Aghlmandi S., Dalcanale F., Seiler D., Zeilhofer H.-F., Honigmann P., Thieringer F.M. (2021). Quantitative Assessment of Point-of-Care 3D-Printed Patient-Specific Polyetheretherketone (PEEK) Cranial Implants. Int. J. Mol. Sci..

[43] Skirbutis G., Dzingutė A., Masiliūnaitė V., Šulcaitė G., Žilinskas J. (2017). A Review of PEEK Polymer’s Properties and Its Use in Prosthodontics. Stomatologija.

[44] Mishra S., Chowdhary R. (2019). PEEK Materials as an Alternative to Titanium in Dental Implants: A Systematic Review. Clin. Implant. Dent. Relat. Res..

[45] Polyetheretherketone (PEEK): MakeItFrom.com. https://www.makeitfrom.com/material-properties/Polyetheretherketone-PEEK.

[46] Papathanasiou I., Kamposiora P., Papavasiliou G., Ferrari M. (2020). The Use of PEEK in Digital Prosthodontics: A Narrative Review. BMC Oral Health.

[47] Kurtz S.M., Devine J.N. (2007). PEEK Biomaterials in Trauma, Orthopedic, and Spinal Implants. Biomaterials.

[48] Ma H., Suonan A., Zhou J., Yuan Q., Liu L., Zhao X., Lou X., Yang C., Li D., Zhang Y. (2021). PEEK (Polyether-Ether-Ketone) and Its Composite Materials in Orthopedic Implantation. Arab. J. Chem..

[49] Garrido B., Albaladejo-Fuentes V., Cano I.G., Dosta S. (2022). Development of Bioglass/PEEK Composite Coating by Cold Gas Spray for Orthopedic Implants. J. Spray Tech..

[50] Yadav D., Garg R.K., Ahlawat A., Chhabra D. (2020). 3D Printable Biomaterials for Orthopedic Implants: Solution for Sustainable and Circular Economy. Resour. Policy.

[51] Alqurashi H., Khurshid Z., Syed A.U.Y., Rashid Habib S., Rokaya D., Zafar M.S. (2021). Polyetherketoneketone (PEKK): An Emerging Biomaterial for Oral Implants and Dental Prostheses. J. Adv. Res..

[52] Qin L., Yao S., Zhao J., Zhou C., Oates T.W., Weir M.D., Wu J., Xu H.H.K. (2021). Review on Development and Dental Applications of Polyetheretherketone-Based Biomaterials and Restorations. Materials.

[53] Alexakou E., Damanaki M., Zoidis P., Bakiri E., Mouzis N., Smidt G., Kourtis S. (2019). PEEK High Performance Polymers: A Review of Properties and Clinical Applications in Prosthodontics and Restorative Dentistry. Eur. J. Prosthodont. Restor. Dent..

[54] Bathala L., Majeti V., Rachuri N., Singh N., Gedela S. (2019). The Role of Polyether Ether Ketone (Peek) in Dentistry—A Review. J. Med. Life.

[55] Van de Vijfeijken S.E.C.M., Münker T.J.A.G., Spijker R., Karssemakers L.H.E., Vandertop W.P., Becking A.G., Ubbink D.T. (2018). CranioSafe Group Autologous Bone Is Inferior to Alloplastic Cranioplasties: Safety of Autograft and Allograft Materials for Cranioplasties, a Systematic Review. World Neurosurg..

[56] Binhammer A., Jakubowski J., Antonyshyn O., Binhammer P. (2020). Comparative Cost-Effectiveness of Cranioplasty Implants. Plast Surg.

[57] Kinsman M., Aljuboori Z., Ball T., Nauta H., Boakye M. (2020). Rapid High-Fidelity Contour Shaping of Titanium Mesh Implants for Cranioplasty Defects Using Patient-Specific Molds Created with Low-Cost 3D Printing: A Case Series. Surg Neurol. Int..

[58] MedCAD | PEEK Cost and Price Comparison. https://medcad.com/peek-cost-and-price-comparison/.

[59] Thien A., King N.K.K., Ang B.T., Wang E., Ng I. (2015). Comparison of Polyetheretherketone and Titanium Cranioplasty after Decompressive Craniectomy. World Neurosurg..

[60] Wyleżoł M. (2018). Hybrid Modeling Methods of Cranial Implants. Adv. Sci. Technol. Res. J..

[61] Al-Ahmari A., Nasr E.A., Moiduddin K., Anwar S., Kindi M.A., Kamrani A. (2015). A Comparative Study on the Customized Design of Mandibular Reconstruction Plates Using Finite Element Method. Adv. Mech. Eng..

[62] High-Performance Materials. https://www.intamsys.com/high-performance-materials/.

[63] Marcián P., Narra N., Borák L., Chamrad J., Wolff J. (2019). Biomechanical Performance of Cranial Implants with Different Thicknesses and Material Properties: A Finite Element Study. Comput. Biol. Med..

[64] Ridwan-Pramana A., Marcián P., Borák L., Narra N., Forouzanfar T., Wolff J. (2017). Finite Element Analysis of 6 Large PMMA Skull Reconstructions: A Multi-Criteria Evaluation Approach. PLoS ONE.

[65] Brandicourt P., Delanoé F., Roux F.-E., Jalbert F., Brauge D., Lauwers F. (2017). Reconstruction of Cranial Vault Defect with Polyetheretherketone Implants. World Neurosurg..

[66] Kim K., Noh H., Park K., Jeon H.W., Lim S. (2022). Characterization of Power Demand and Energy Consumption for Fused Filament Fabrication Using CFR-PEEK. Rapid Prototyp. J..

[67] Jiang C.-P., Cheng Y.-C., Lin H.-W., Chang Y.-L., Pasang T., Lee S.-Y. (2022). Optimization of FDM 3D Printing Parameters for High Strength PEEK Using the Taguchi Method and Experimental Validation. Rapid Prototyp. J..

[68] Lalegani Dezaki M., Mohd Ariffin M.K.A., Hatami S. (2021). An Overview of Fused Deposition Modelling (FDM): Research, Development and Process Optimisation. Rapid Prototyp. J..

[69] Mahendru S., Jain R., Aggarwal A., Aulakh H.S., Jain A., Khazanchi R.K., Sarin D. (2020). CAD-CAM vs Conventional Technique for Mandibular Reconstruction with Free Fibula Flap: A Comparison of Outcomes. Surg. Oncol..

[70] Nayman J., Pearson E.S. (1933). On the Problem of the Most Efficient Tests of Statistical Hypotheses. Phil. Trans. R. Soc. Lond. A.

[71] Lo Giudice A., Ronsivalle V., Grippaudo C., Lucchese A., Muraglie S., Lagravère M.O., Isola G. (2020). One Step before 3D Printing—Evaluation of Imaging Software Accuracy for 3-Dimensional Analysis of the Mandible: A Comparative Study Using a Surface-to-Surface Matching Technique. Materials.

[72] Geomagics Control. X; 3D Systems. https://www.3dsystems.com/software#inspectionsoftware.

[73] Hammad Mian S., Abdul Mannan M., Al-Ahmari A. (2014). The Influence of Surface Topology on the Quality of the Point Cloud Data Acquired with Laser Line Scanning Probe. Sens. Rev..

[74] Verbruggen S.W., Vaughan T.J., McNamara L.M. (2012). Strain Amplification in Bone Mechanobiology: A Computational Investigation of the in Vivo Mechanics of Osteocytes. J. R Soc. Interface.

[75] Carter D.R., Fyhrie D.P., Whalen R.T. (1987). Trabecular Bone Density and Loading History: Regulation of Connective Tissue Biology by Mechanical Energy. J. Biomech.

[76] Mosley J.R. (2000). Osteoporosis and Bone Functional Adaptation: Mechanobiological Regulation of Bone Architecture in Growing and Adult Bone, a Review. J. Rehabil Res. Dev..

[77] Santos P.O., Carmo G.P., de Sousa R.J.A., Fernandes F.A.O., Ptak M. (2022). Mechanical Strength Study of a Cranial Implant Using Computational Tools. Appl. Sci..

